# MultiResUNet3+: A Full-Scale Connected Multi-Residual UNet Model to Denoise Electrooculogram and Electromyogram Artifacts from Corrupted Electroencephalogram Signals

**DOI:** 10.3390/bioengineering10050579

**Published:** 2023-05-10

**Authors:** Md Shafayet Hossain, Sakib Mahmud, Amith Khandakar, Nasser Al-Emadi, Farhana Ahmed Chowdhury, Zaid Bin Mahbub, Mamun Bin Ibne Reaz, Muhammad E. H. Chowdhury

**Affiliations:** 1 Department of Electrical, Electronic and Systems Engineering, Universiti Kebangsaan Malaysia, Bangi 43600, Malaysia; 2 Department of Electrical Engineering, Qatar University, Doha 2713, Qatar; 3 Department of Electronics and Telecommunication Engineering, Rajshahi University of Engineering and Technology, Rajshahi 6204, Bangladesh; 4 Department of Mathematics and Physics, North South University, Dhaka 1229, Bangladesh; 5 Department of Electrical and Electronic Engineering, Independent University, Bashundhara, Dhaka 1229, Bangladesh

**Keywords:** electroencephalogram (EEG), electrooculogram (EOG), electromyogram (EMG), artifacts, denoising, MultiResUNet3+, 1D-CNN, deep learning

## Abstract

Electroencephalogram (EEG) signals immensely suffer from several physiological artifacts, including electrooculogram (EOG), electromyogram (EMG), and electrocardiogram (ECG) artifacts, which must be removed to ensure EEG’s usability. This paper proposes a novel one-dimensional convolutional neural network (1D-CNN), i.e., MultiResUNet3+, to denoise physiological artifacts from corrupted EEG. A publicly available dataset containing clean EEG, EOG, and EMG segments is used to generate semi-synthetic noisy EEG to train, validate and test the proposed MultiResUNet3+, along with four other 1D-CNN models (FPN, UNet, MCGUNet, LinkNet). Adopting a five-fold cross-validation technique, all five models’ performance is measured by estimating temporal and spectral percentage reduction in artifacts, temporal and spectral relative root mean squared error, and average power ratio of each of the five EEG bands to whole spectra. The proposed MultiResUNet3+ achieved the highest temporal and spectral percentage reduction of 94.82% and 92.84%, respectively, in EOG artifacts removal from EOG-contaminated EEG. Moreover, compared to the other four 1D-segmentation models, the proposed MultiResUNet3+ eliminated 83.21% of the spectral artifacts from the EMG-corrupted EEG, which is also the highest. In most situations, our proposed model performed better than the other four 1D-CNN models, evident by the computed performance evaluation metrics.

## 1. Introduction

The electrophysiological activity of the cerebral cortex of the human brain is represented by electroencephalogram (EEG) signals, which are non-invasively recorded at the scalp [1]. EEG is crucial for various therapeutic applications as well as neurological research. Long-duration human epileptic seizure episodes are routinely detected with EEG [2,3]. EEG is also widely used for other purposes, such as Alzheimer’s disease diagnosis [4,5], sleep stages measurement [6], assessment of cognitive workload [7], recognition of human emotion [8], establishing brain-computer interfaces (BCIs) [9], biometric systems [10], etc. EEG recordings, nevertheless, greatly suffer from a range of physiological and non-physiological artifacts, such as ocular/electrooculogram (EOG) artifacts [11], myogenic/electromyogram (EMG) noises [12], cardiac/electrocardiogram (ECG) abnormalities [13,14], power line noise and motion artifacts [15,16,17]. These noises will significantly impact the EEG data analysis outcome and may even lead to an entirely wrong diagnosis in the worst cases. To remove the physiological artifacts and maintain the neural information of EEG as much as possible, it is required to develop efficient, reliable, and robust algorithms/models.

One frequently used strategy to remove artifacts from EEG data involved converting the signal from the time domain to the frequency domain using the Fourier Transform (FT) and filtering out the spectral components corresponding to the artifacts. After discarding the noise component/s and taking the inverse FT, a noise-free signal can be obtained. EEG denoising was also carried out using filters, including the adaptive, Wiener, and Kalman filters [18,19,20]. Other signal processing-based techniques, such as the Hilbert–Huang Transformation (HHT) [21,22], empirical mode decomposition (EMD) [23,24], variational mode decomposition (VMD) [16], wavelet packet decomposition (WPD) [17], independent component analysis (ICA) [25,26], and canonical correlation analysis (CCA) [27], etc., were also utilized. These methods primarily relied on linear transformation and several presumptions, which is a severe limitation—particularly, capturing artifactual signals as the reference is an essential requirement for all types of adaptive filtering. Therefore, the denoising performance could be relatively subpar without the reference signal. The HHT-based artifact removal technique makes the false assumption that the noisy signals or components encoded in EEG have specific time properties from the other components. In [22,28], via an adaptable manner, the EEG signals were decomposed into intrinsic mode functions (IMFs), and the instantaneous frequency of the IMF was computed using HHT. The IMFs with significant deviations from other IMFs in terms of instantaneous frequency were discarded, considering them as artifact components. In [29,30], EEG signals were divided into several modes for both EMD-based and ICA-based techniques, and the noise-related components were subsequently removed using some predetermined criteria. The elimination of EMG artifacts typically involved CCA-based EEG denoising techniques, assuming that the muscle artifacts had infrequent stereotyped topographies and low autocorrelation. The muscle artifacts to be eliminated were determined by the CCA, which decomposes the EEG data into two or more uncorrelated components [15]. Some hybrid techniques like Ensemble EMD (EEMD) in combination with ICA (EEMD-ICA) [31] and EEMD-CCA [32] were investigated to remove EEG noises. However, the necessity of prior assumptions remains unresolved despite performance boosts compared to other conventional approaches. As an illustration, the value of autocorrelation thresholds in the EEMD-CCA needs to be chosen empirically through trial and error [33].

Recently, deep learning (DL) has garnered increasing interest. The effectiveness of DL-based models has significantly improved because of the growth of computing resources, the continuous development of novel network designs, and the processing capability of vast amounts of data, etc. DL has been successfully used to address various technical issues, including image processing [34,35,36] and natural language processing [37,38,39]. EEG-based motor imagery classification [40,41,42], EEG reconstruction [43,44], and EEG signal creation [45,46] are some noteworthy examples of EEG-related analysis where DL techniques have been utilized. Some established DL models, such as auto-encoder [47,48], simple and complex convolutional neural networks (CNNs) with/without residual connections [49,50], recurrent neural networks (RNNs) [50,51], and generative adversarial networks (GANs) [52], have also been used to remove artifacts from contaminated EEG. To disentangle neural signals from artifacts in the embedding space and recreate the noise-free signal, a new deep learning framework called DeepSeparator has been introduced [53]. Although significant progress has been made in the denoising of EEG through the utilization of DL-based methods, there are still some drawbacks in the studies reported earlier, such as lack of robust framework, non-usage of k-fold cross-validation methods, testing with a selective portion of the dataset, lack of model’s generalizability, the potential for further performance improvements, and the lack of appropriate performance metrics to evaluate the models.

This work is carried out with the motivation of implementing a robust and reliable DL-based model capable of removing major physiological artifacts (EOG/EMG/simultaneous EOG and EMG) from the contaminated EEG signals with improved performance. In this light, a novel one-dimensional (1D) segmentation network (MultiResUNet3+) to denoise (i) ocular/EOG, (ii) myogenic/EMG, and (iii) simultaneous EOG and EMG artifacts from contaminated EEG is proposed, and the efficacy of the MultiResUNet3+ model is compared with four other 1D-CNN models, namely (i) Feature Pyramid Network (FPN) [54], (ii) UNet [55], (iii) MCGUNet [56], and (iv) LinkNet [57]. Semi-synthetic EEG segments were generated from a publicly available dataset for training and testing DL-based 1D-CNN models [50]. The quantitative and qualitative findings from the proposed MultiResUNet3+, along with four other 1D-CNN models, provide strong evidence of the capacity of DL models to acquire knowledge of the intrinsic characteristics of artifacts mixed into EEG data, detect them, and remove/reduce them reliably and efficiently. The following are our primary contributions, in brief:

- The proposed MultiResUNet3+ can effectively denoise EOG, EMG, and concurrent EOG and EMG artifacts from corrupted EEG waveforms.
- We have created a diverse and representative semi-synthetic EEG dataset closely resembling real-world corrupted EEG signals. The proposed 1D-segmentation model was trained and evaluated using 5-fold cross-validation, which ensured the reliability and robustness of the proposed model.
- We used five well-established performance metrics to comprehensively assess and compare the denoising performance of each of the five 1D-segmentation models.
- Our developed model may be used for denoising multi-channel, actual EEG data as the model was trained with diverse artifactual data.

The remainder of this manuscript has been structured as follows: Section 2 explains the intricacies of the novel MultiResUNet3+ segmentation network’s architecture. This is trailed by a comprehensive overview of the EEG dataset employed, together with the semi-synthetic EEG data generation and normalization approaches that have been adopted in this study. Section 3 provides the experimental details, explaining the formulae of the performance metrics that are readily available for usage. The quantitative and qualitative performance of the proposed model compared with four different 1D-CNN models is provided in Section 4, and an analysis of the results is presented. Section 5 comprises a cogent discussion of the outcomes, including the limitations inherent in our study, while the paper is concluded concisely in Section 6.

## 2. Materials and Methods

**Figure 1:**
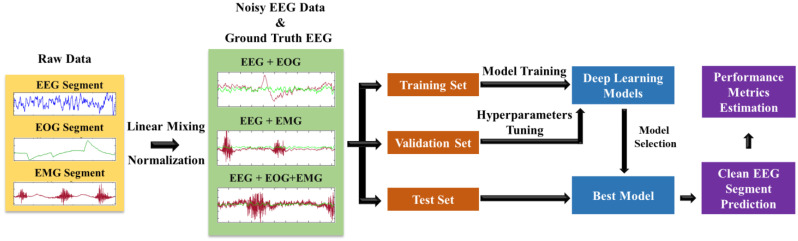
Proposed framework for removing physiological artifacts from EEG signals.

Using the most up-to-date 1D-CNN-based segmentation networks, Figure 1 shows the end-to-end framework suggested in this study for efficiently denoising EEG signals semi-synthetically corrupted by EOG/EMG/concurrent EOG and EMG artifacts. The figure provides a comprehensive visual representation of our proposed approach and requires no further elucidation.

In the subsequent sub-sections, we detail our proposed MultiResUNet3+ segmentation network, which forms the core of our denoising framework. We also present a concise overview of the EEG dataset employed in this work and discuss the semi-synthetic data generation and normalization techniques adopted in our study, which are crucial in achieving reliable and robust denoising results.

### 2.1. Proposed Novel MultiResUNet3+ Model Description

**Figure 2:**
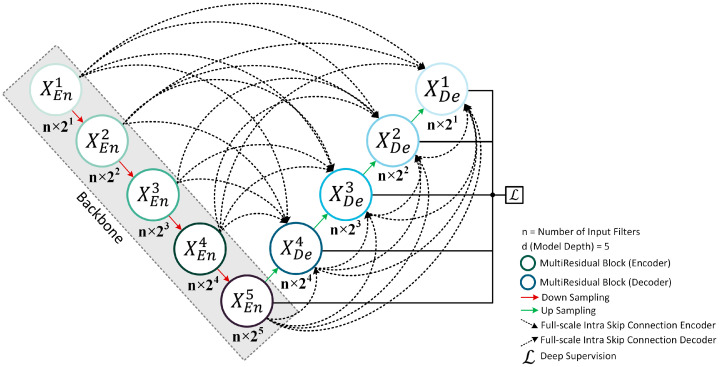
The proposed MultiResUNet3+ segmentation model architecture.

Our proposed MultiResUNet3+ model for segmentation effectively combines the concepts of MultiResUNet [58] and UNet3+ [59] networks inside a single framework. The building blocks of the 1D-MultiResUNet3+ model used in this study (depth of 5) have been depicted in Figure 2.

**Figure 3:**
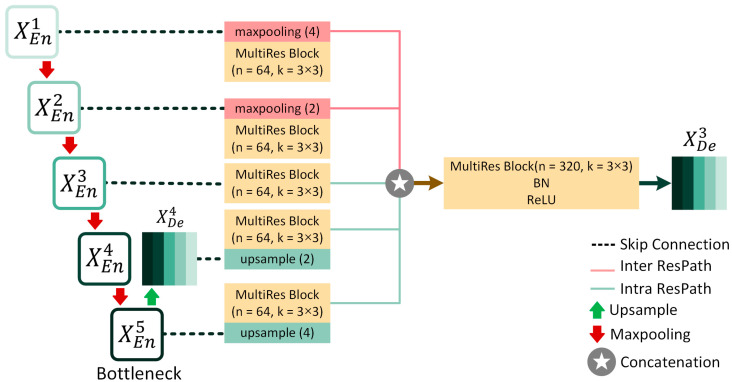
Full-Scale Aggregated Feature Map Creation for the third decoder Layer $X_{De}^{3}$ aided by Residual Paths (ResPaths in short), following the process proposed in [59].

**Figure 4:**
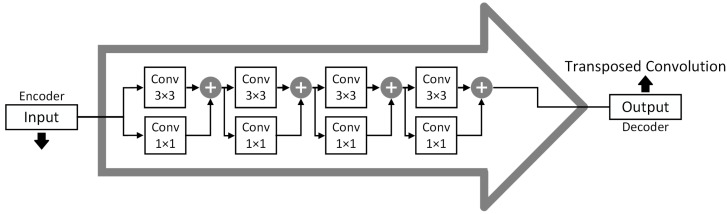
Residual Path architecture adapted from [58] used for inter- and intra-connections in MultiResUNet3+ is expected to reduce the semantic gaps by replacing direct skip connections. This particular ResPath represents the one produced from $X_{En}^{1}$ and $X_{De}^{2}$ among the inter- and intra-ResPaths, respectively, in Figure 2.

UNet3+ consists of full-scale skip connections combining interconnections between the encoders and the decoders and intra-connections between the decoder sub-networks. Unlike UNet [55] and UNet++ [60], UNet3+ can incorporate small and larger-scale feature maps in each decoder layer, allowing it to extract fine and coarse-grained semantics in full scales. It was employed in the MultiResUNet3+ for this application to effectively minimize semantically different EOG and EMG artifacts from EEG waveforms. EMG requires a finer approach, while EOG-induced impurities are coarser. Nevertheless, UNet3+ still used direct skip connections for inter- and intra-connections, prevailing the semantic gap issue in the basic UNet architecture, which was solved by more advanced architectures, such as UNet++ and MultiResUNet. To reduce the semantic gap, MultiResUNet or similar architectures proposed replacing the skip connections with various formations of convolutional blocks. Reducing the semantic gaps in this application will help the model efficiently learn and generate EEG features, especially when EOG and EMG noises are mixed, to create a more realistic but challenging scenario. Therefore, contrary to UNet3+, our proposed MultiResUNet3+ contains full-scale Residual Paths (Figure 3) instead of direct skip connections for inter- and intra-connections. Instead of combining the encoder and decoder features straightforwardly (e.g., concatenation in UNet and addition in LinkNet [57]), the encoder features are passed through several convolutional layers with residual connections (Figure 4). Mentionable that residual connections [35] have been beneficial in several deep learning applications during their learning process. The depth of the network affects how many residual-convolutional blocks are used in the inter- and intra-ResPaths for MultiResUNet3+. For example, for the model drawn in Figure 2 with a depth of 5, the number of residual-convolutional blocks along the inter-ResPaths will be 4, 3, 2, and 1, respectively, generated from shallower to deeper layers. On the other hand, for the intra-ResPaths densely connecting the decoders, the number of residual-convolutional blocks will be 1, 2, 3, and 4, respectively, from deeper to shallower layers. More residual-convolutional blocks are placed along the ResPaths generated from a shallower layer for improved processing of the coarser features.

Here, Figure 3 represents the full-scale aggregated feature map creation process for MultiResUNet3+ for the decoder $X_{De}^{3}$. Similar to MultiResUNet, the feature map from the same scale encoder layer $X_{En}^{3}$ is received by the decoder following a ResPath. However, contrary to MultiResUNet, a set of encoder-decoder Residual Paths delivers the semantically enhanced low-level detailed information from the smaller-scale encoder layers $X_{En}^{1}$ and $X_{En}^{2}$ through non-overlapping max-pooling operations.

**Figure 5:**
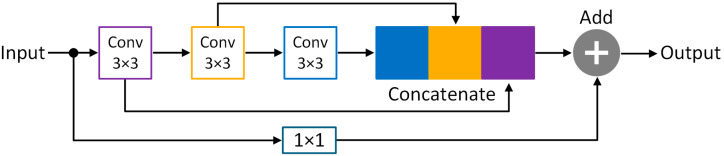
Structure of the Modified MultiResidual or MultiRes Block inspired from [58].

On the other hand, higher-level semantic information is conveyed from the larger-scale decoder layers, such as $X_{De}^{4}$ and $X_{De}^{5}$, through intra-ResPath connections by utilizing nearest interpolation. Now, as in Figure 2 and Figure 3, there are five total inter- and intra-Residual Paths (Figure 4) containing same-resolution feature maps waiting to be unified before reaching $X_{De}^{3}$. To properly merge the shallow and deep semantic information, we perform a feature aggregation mechanism on the ResPaths by placing a Multi-Residual or MultiRes Block (Figure 5) containing W=n×d input kernels or filters of size 3×3 after concatenating the five feature maps. Here, *n* denotes the input filter number, and the depth of the model is represented by *d*. For example, if *n* is 64 and *d* is 5, *W* will be 320.

The MultiRes Block used in MultiResUNet3+ is similar to the “Module A” of the InceptionV4 network [61]. It consists of a succession of 3×3 sized filters being concatenated and then element-wise added by a residual connection passed through a 1×1 convolutional block to squeeze dimensions. This helps us to aggregate spatial features from various context sizes while avoiding expensive filters with larger kernels (e.g., 5×5 or 7×7, primarily used in earlier Inception networks). For the MultiRes block, instead of keeping the number of filters the same, it is more efficient and less expensive to gradually increase them while keeping the total number of filters the same as if it were a single convolutional block, as explained in [58]. For instance, if the number of input filters for the MultiRes block used for feature aggregation is W=n×d=320, the width of the three convolution blocks inside the MultiRes block will be w/6=round(320/6)≈53, $\frac{W}{3}=round(320/3)\approx 107$, and w/2=160, respectively. Each MultiRes block is followed by batch normalization and ReLU activation layers.

### 2.2. Dataset Description

A publicly available dataset, namely EEGdenoiseNet [50], was found to be suitable for this work as it contained pre-processed, well-structured physiological segments gathered from various sources, including 4514 clean EEG segments, 3400 pure EOG segments, and 5598 clean EMG segments. We have used these readily available segments to generate semi-synthetic noisy EEG, which were used to train and test the five distinct 1D-segmentation models.

**Figure 6:**
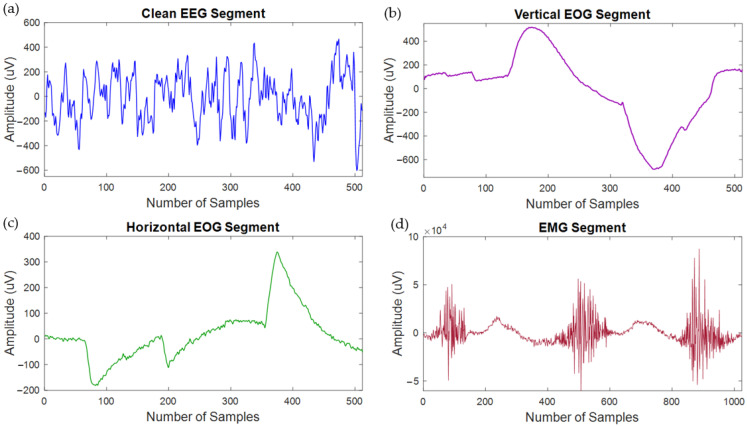
Sample (**a**) clean or ground truth EEG, (**b**) pure vertical EOG, (**c**) noise-free horizontal EOG, and (**d**) clean EMG segment with actual amplitudes. Noticeably, EMG has a much higher magnitude compared to clean EEG and EOG segments.

Several signal-processing techniques were adopted by the EEGdenoiseNet [50] authors. They processed the clean EEG signals (collected from several sources) by applying a bandpass filter with the lower and upper cutoff frequencies of 1 and 80 Hz, removed the power line noise using a notch filter, and then down-sampled at 256 Hz. The clean EEG signals were then segmented, and 4514 clean EEG segments were generated, each containing 512 data points. The clean EOG signals (horizontal and vertical) were bandpass filtered with a passband of 0.3–10 Hz, resampled to 256 data points per second. These processed clean EOG signals were segmented, and 3400 segments were produced, where each clean EOG segment has 512 data points, the same as the EEG segments. The EMG signal was filtered using a bandpass filter with a lower cutoff frequency of 1 Hz and upper cutoff frequency of 120 Hz, and the powerline frequency component was removed using a notch filter. Then the raw EMG signals were resampled to 512 data points per second, and 5598 clean muscle/EMG segments were generated, each containing 1024 data points [50]. As EMG signals have essential features in the high-frequency band, the clean EMG signals were sampled at 512 Hz to retain those features and morphology. Figure 6 depicts one clean EEG, one clean vertical EOG, one pure horizontal EOG, and one clean EMG segment sample. Since the authors of [50] adopted all the necessary pre-processing techniques, no further pre-processing steps were undertaken in this study. Instead, the publicly available segments are directly used to produce a large and diverse semi-synthetic corrupted EEG dataset.

### 2.3. Semi-Synthetic Electroencephalogram Segment Generation and Normalization

For this study, we generate semi-synthetic EOG, EMG, and EOG-EMG contaminated EEG segments from the true signals in the EEGdenoiseNet dataset. By linearly mixing one clean EEG segment with one clean EOG and/or EMG segment using Equation (1), a semi-synthetic corrupted EEG segment can be produced [50]:

(1) y=x+λ·n

Here, x denotes the ground truth or clean EEG segments, y is the generated semi-synthetic noisy EEG, and n characterizes the EOG and/or EMG artifacts. By solving Equation (2) and altering the scaling factor λ, the signal-to-noise Ratio (SNR) of the semi-synthetically generated contaminated EEG segment can easily be adjusted to different levels as follows [50]:

(2) $$SNR=10\;log\frac{RMS(x)}{RMS(\lambda \cdot n)}$$

For the computation of root mean square (RMS) value, Equation (3) is utilized [50]:

(3) $$RMS\left(w\right)=\sqrt{\frac{1}{m}\sum_{i=1}^{m}w_{i}^{2}}$$

Here, m stands for the total number of data points of segment w, and $w_{i}$ stands for the $i_{th}$ sample point of w. The scaling factor λ is pivotal in determining the SNR of the semi-synthetic, corrupted EEG segments. As a rule of thumb, a lower value of λ corresponds to a higher SNR, whereas a higher λ leads to a poorer SNR. The SNR of EEG solely corrupted by EOG artifacts typically falls within the range of −7 to 2 dB, as reported in previous studies [23]. On the other hand, EEG signals corrupted by EMG artifacts exhibit a comparatively wider range of SNR, typically ranging from −7 to 4 dB [62]. The availability of ground truth and noisy EEG segments allowed us to train, test and validate the deep learning models for EEG denoising. However, rather than directly feeding the (x,y) pairs into the 1D-CNN models, we considered the standard deviation of the corrupted EEG segments ($\sigma_{y}$) and divided the clean and contaminated EEG signals by this value (Equation (4)). The result was a pair of rescaled or normalized segments ($\hat{x},\hat{y}$), fed to the 1D-segmentation models for training, testing, and validation.

(4) $$\hat{x}=\frac{x}{\sigma_{y}};\;\hat{y}=\frac{y}{\sigma_{y}}$$

Since deep learning models are sensitive to variations in the magnitude of input data, without scaling or normalization, the model may be unable to make useful inferences. Normalization aids in keeping all input features of equal importance, which is especially helpful when dealing with large-scale data variations. Normalization also helps the model to converge faster during model training by minimizing the variance of the input data. Moreover, the training process can be accelerated via normalization, and the model’s learning capacity can be facilitated. Again, normalization aids in avoiding overfitting.

In this study, the EOG-contaminated EEG segments were generated by linearly mixing randomly chosen 3400 clean EEG segments (out of 4514 clean EEG segments) with the available 3400 EOG segments. This process was repeated for ten different integer SNR levels (−7 to 2 dB), producing 34,000 semi-synthetic EOG-contaminated EEG segments. The sampling frequency of both EOG and EEG segments was kept at 256 Hz. It is worthwhile to mention that as there were 4514 clean EEG segments available, we could easily produce 45,140 semi-synthetic corrupted EEG segments for ten different SNR levels by using some clean EOG more than once during linear mixing. However, this process was avoided to prevent data-leaking issues.

**Figure 7:**
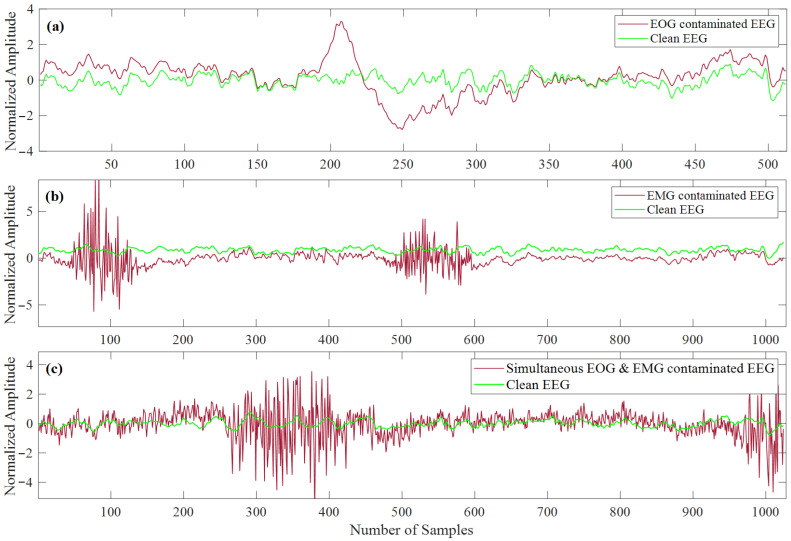
Superimposed arbitrary example segments of (**a**) EOG-corrupted EEG and corresponding ground truth EEG, (**b**) EMG-contaminated EEG and corresponding clean EEG, (**c**) simultaneous EOG and EMG-corrupted EEG with corresponding ground truth EEG.

Similarly, EMG-contaminated EEG segments were created by combining the EEG segments with the randomly selected clean EMG segments for 10 different SNR levels. Before linearly mixing EEG and EMG segments, all the clean EEG segments were up-sampled by a factor of 2 to match the number of data points each EMG segment contains. The upsampling of EEG (from 256 to 512 Hz) did not cause any morphological change in EEG segments as the bandwidth of EEG is 1–80 Hz [63]. Eventually, we generated 45,140 EMG-contaminated EEG segments for the ten different SNR levels semi-synthetically. The simultaneous EOG and EMG contaminated EEG segments were generated by mixing the 3400 clean EEG segments (randomly taken out from 4514 clean EEG segments) with 3400 EOG and 3400 EMG (randomly taken out of 5598 EMG segments) segments for ten different SNR levels. The clean EEG and EOG segments were up-sampled at 512 Hz before linear mixing to match the sampling frequency of the EMG segments. This process generated 34,000 corrupted EEG segments for all ten different SNR levels. Figure 7a displays a picked at-random EOG-contaminated EEG segment. Figure 7b illustrates one arbitrary EMG-contaminated EEG segment, whereas Figure 7c represents one random sample of simultaneous EOG and EMG-contaminated EEG segments. The corresponding ground truth EEG segments are also superimposed in Figure 7a–c.

## 3. Experimental Setup

In pursuit of denoising EEG signals from physiological (EOG/EMG/concurrent EOG and EMG) artifacts, the novel MultiResUNet3+ model, alongside four other 1D-CNN models, was trained. The training involved feeding the neural networks normalized contaminated EEG segments as input (generated using Equation (4)) while providing corresponding normalized ground truth EEG segments as output. This process made it easier for the DL-based model to create a nonlinear function that translated the noisy EEG to its equivalent ground truth. The mean squared error (MSE) was employed as the loss function to generate the nonlinear mapping function. The loss function was optimized using the Adam optimizer with a learning rate of 0.0005. Eighty percent of the data was used as the training set and the remaining 20% for the test set. Ten percent of the training set data were used as the validation set. It is worth mentioning here that each EEG, EOG, and EMG segment is only used once to produce the semi-synthetic dataset. Therefore, any sort of data leaking between train, test, and validation sets is absent. All five networks were trained, validated, and tested independently using the five-fold cross-validation technique in the Google ColabPro environment using Python 3.10 framework to ensure robustness and reliability in the evaluation process. In this work, the experimentation was done with a twofold approach which is described below:

### 3.1. Experiment A

As mentioned earlier, there were in total 34,000 EOG-contaminated EEG segments having ten integer SNR values ranging from −7 to +2 dB, where for each SNR value, 3400 EEG segments contaminated with ocular artifacts were generated. For EMG-contaminated EEG, the total number of contaminated segments was 45,140. In contrast, for simultaneous EOG and EMG corrupted EEG, this number was 34,000 for ten different SNR levels ranging from −7 to +2 dB. For all these three types of artifacts, we trained each of the five 1D-CNN models ten times, utilizing the corrupted and corresponding ground truth segments for ten different SNR values. That is, for EOG corrupted EEG segments at SNR level −7 dB, 80% of 3400 segments (2720) were used for training the models, and the remaining 20% (680 segments) were utilized for testing. This same process was carried out separately for nine other integer SNR levels (−6 to 2 dB).

Similarly, for ten different SNR levels, EMG-contaminated EEG segments, and simultaneous EOG and EMG-contaminated EEG segments, were utilized for training and testing all five networks ten times separately. Employing a deep supervision technique as described in [64], in this experiment, we computed three established performance metrics, namely the correlation coefficient (CC) in the time domain, and temporal and spectral relative root mean squared error (RRMSE). These metrics were particularly used to evaluate the effectiveness of the five DL-based models in denoising contaminated EEG. The five-fold cross-validation technique was employed, and the metrics mentioned earlier were computed for each of the five folds to ensure robustness and reliability in the evaluation process.

### 3.2. Experiment B

In Experiment B, a comprehensive approach was taken to generate train and test sets so that a more robust DL-based model could be trained that would perform well across a wide range of SNR levels, which is crucial for real-world applications where the exact SNR of the input signal may vary due to factors, such as electrode placement, the patient’s movements, or equipment quality. For each of the 10 different SNR levels, 80% of the EOG-contaminated EEG segments and their corresponding ground truth EEG segments were extracted to produce a more extensive dataset (2720 pairs in each SNR level). After merging these pairs, a training dataset of 27,200 pairs was created. The remaining 20% of pairs from each of the ten distinct SNR levels were used to generate the test set (6800 pairs). Unlike in Experiment A, where models were trained and evaluated for specific SNR level segments, the models in Experiment B were trained using combined segments of all ten distinct SNR levels. This improved model generalization, making them more resilient when evaluated with noisy EEG segments with varying SNR levels. In essence, Experiment B took a holistic approach to train DL models that could perform well across the noisy EEG segments having a range of SNR levels, thereby increasing their utility in real-world scenarios where the SNR of the input signal may not be precisely known. Following a similar protocol, a total of 36,110 EMG-contaminated EEG segments and 27,200 pairs of simultaneous EOG and EMG-contaminated EEG segments were generated separately to form the train set, and the remaining 20% contaminated EEG segments were used as test set (9030 pairs of EMG-contaminated EEG segments, and 6800 pairs of simultaneous EOG and EMG contaminated EEG segments) for the segmentation models. The efficacy of the trained models was quantitatively measured using five performance metrics, i.e., the percentage reduction in artifacts in the time and frequency domain, temporal and spectral RRMSE, and the average power ratio of each of the five different EEG bands (Alpha, Beta, Gamma, Delta, Theta bands) to the whole band separately.

### 3.3. Performance Evaluation Metrics

The proper quantitative assessment of any DL model is paramount in determining its ability to accomplish the intended task, in our case, effectively denoising EEG signals. In this study, meticulous attention was devoted to selecting the most appropriate performance parameters for an adept evaluation of the chosen segmentation models. The efficacy of the five 1D-CNN models was quantitatively evaluated by calculating several temporal and spectral performance metrics, such as the correlation coefficient (CC), the percentage reduction in artifacts, and the relative root mean squared error (RRMSE). These metrics were critical in determining the best-performing models. The relevant equations for these measures can be found in Equations (5)–(9), as collected from [50,65],

(5) $${CC}_{temporal}=\frac{Cov(\hat{z},\hat{x})}{\sqrt{Var(\hat{z})Var(\hat{x})}}$$

(6) $$\eta =\left(1-\frac{1-{CC}_{temporal\left(af\right)}}{1-{CC}_{temporal\left(bf\right)}}\right)*100$$

(7) $$\gamma =\left(1-\frac{1-{CC}_{spectral\left(af\right)}}{1-{CC}_{spectral\left(bf\right)}}\right)*100$$

(8) $${RRMSE}_{temporal}=\frac{RMS(\hat{z}-\hat{x})}{RMS(\hat{x})}$$

(9) $${RRMSE}_{spectral}=\frac{RMS(PSD(\hat{z})-PSD(\hat{x}))}{RMS(PSD\left(\hat{x}\right))}$$

Here, the time domain correlation coefficient is represented by ${CC}_{temporal}$ while covariance is denoted by Cov. The predicted EEG segments are represented by $\hat{z}$, whereas $\hat{x}$ denotes the normalized ground truth EEG segments. Variance is characterized by Var, while η represents the temporal percentage reduction of EOG/EMG/simultaneous EOG and EMG artifacts from the corrupted EEG, and γ represents the percentage reduction of EOG, EMG, or simultaneous EOG and EMG artifacts from the corrupted EEG in the frequency domain. Furthermore, the time domain correlation coefficient between the predicted and the ground truth EEG segments is denoted by ${CC}_{temporal(af)}$, while ${CC}_{temporal(bf)}$ is used to represent the time domain correlation coefficient between contaminated and ground truth EEG segments. Similarly, the frequency domain correlation coefficient between the predicted and the ground truth EEG segments is expressed by ${CC}_{spectral(af)}$, whereas ${CC}_{spectral(bf)}$ is used to represent the frequency domain correlation coefficient between the contaminated and the ground truth EEG segments. RMS is the abbreviation for root mean square, which can be computed using Equation (3), and finally, the PSD is the power spectral density computed using the Periodogram method. The Periodogram method involves calculating the Discrete FT (DFT) of the signal and then taking the square of the absolute value of the DFT to obtain the power spectral density. 

We have selected these performance metrics to evaluate the 1D-CNN-based segmentation models. The reasoning for choosing these metrics is summarized as follows:

The correlation coefficient in the time domain (${CC}_{temporal}$) measures the degree of similarity between two variables. A higher correlation coefficient between predicted and ground truth EEG would indicate that the predicted EEG is more similar to the ground truth EEG and vice-versa. Hence, calculating the correlation coefficient is a valuable tool that quantitatively measures the model’s adeptness/inability in denoising.

The metric temporal percentage reduction in artifacts (η) measures the proportion of EOG/EMG/simultaneous EOG and EMG artifacts removed from the noisy EEG signal in the time domain. A higher temporal percentage reduction in artifacts indicates that more of the artifact has been removed from the EEG signal, resulting in a clean and more accurate EEG signal.

In contrast, the spectral percentage reduction in EOG/EMG/simultaneous EOG and EMG artifacts (γ) measures the proportion of EOG/EMG artifacts removed from the EEG signal in the frequency domain. A higher spectral percentage reduction indicates that more of the artifact has been removed from the EEG signal across all frequency bands and vice-versa.

The RRMSE measured in the time domain (${RRMSE}_{temporal}$) can provide insights into the temporal dynamics of the denoised EEG signals. A low temporal RRMSE value indicates that the predicted individual EEG segment closely approximates the corresponding ground truth EEG segment.

The RRMSE measured in the frequency domain (${RRMSE}_{spectral}$) can help assess the ability of the predictor model to capture important spectral features of the EEG signals in the whole synthetic dataset, such as alpha, beta, gamma, delta, and theta bands. A low spectral RRMSE value indicates that the predicted EEG signal accurately captures the power distribution across different frequency bands. This is important for ensuring that the predicted EEG signal is not biased towards or against any particular frequency band, which could have unintended effects on subsequent analyses.

The average power ratio measures the power of a given frequency band against the power of the EEG signal over the whole spectrum. It is computed by dividing the power of a particular frequency band by the total power of the signal. In general, by calculating the average power ratio for each frequency band separately, we can better understand the distribution of power across the different frequency ranges, which is vital. Specifically, while estimating average power ratios for ground truth and predicted denoised signal for any frequency band, a closely matched numerical value would indicate that the signal is predicted more accurately. In contrast, the power ratio of noisy and ground truth signals should have far apart numerical values. For this reason, the performance of the 1D-CNN models for five different frequency bands of EEG is also reported in this study. Specifically, the five EEG frequency bands are delta (1–4 Hz), theta (4–8 Hz), alpha (8–13 Hz), beta (13–30 Hz), and gamma (30–80 Hz). The average power ratio of each of these bands to the entire band (1–80 Hz) is calculated separately for each predicted, corresponding ground truth, and contaminated EEG segment. The average power ratio was computed following the Periodogram method [65].

## 4. Results

This section details the outcomes of Experiments A and B and analyses the results concisely.

### 4.1. Experiment A Outcomes

For the quantitative performance evaluation of Experiment A, three performance metrics are computed, namely (i) time domain correlation coefficient, (ii) temporal RRMSE, and (iii) spectral RRMSE utilizing the ground truth EEG and the denoised EEG predicted by all five models in three different denoising scenarios, i.e., for EOG-artifacts removal, EMG-artifacts removal, and concurrent EOG and EMG artifacts removal from corrupted EEG, separately.

**Figure 8:**
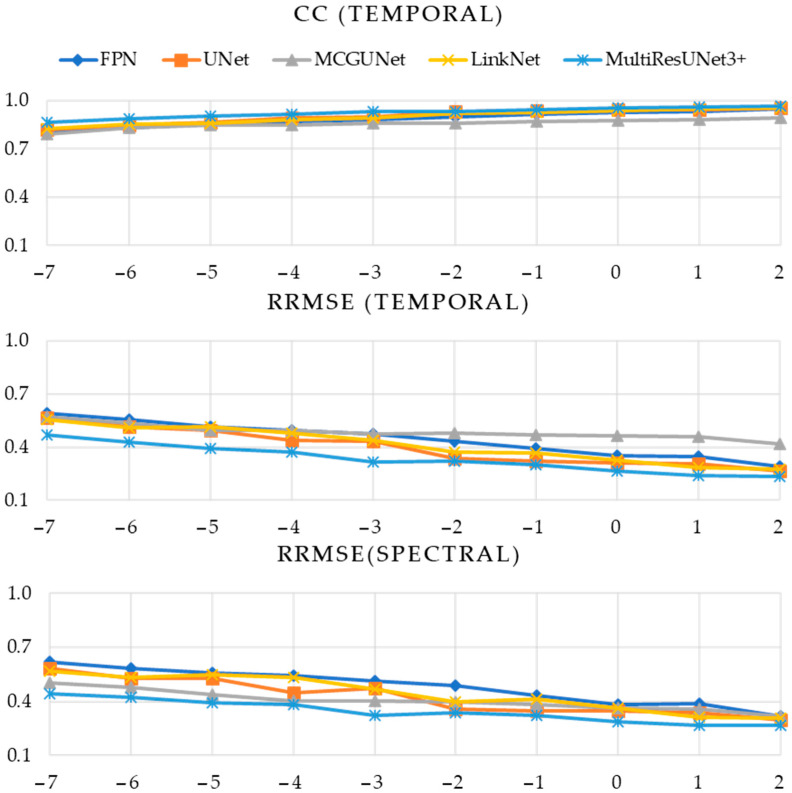
Estimated performance metrics (CC in time domain, RRMSE in time, and frequency domain vs. SNR) of five DL models after denoising EOG-contaminated EEG.

**Figure 9:**
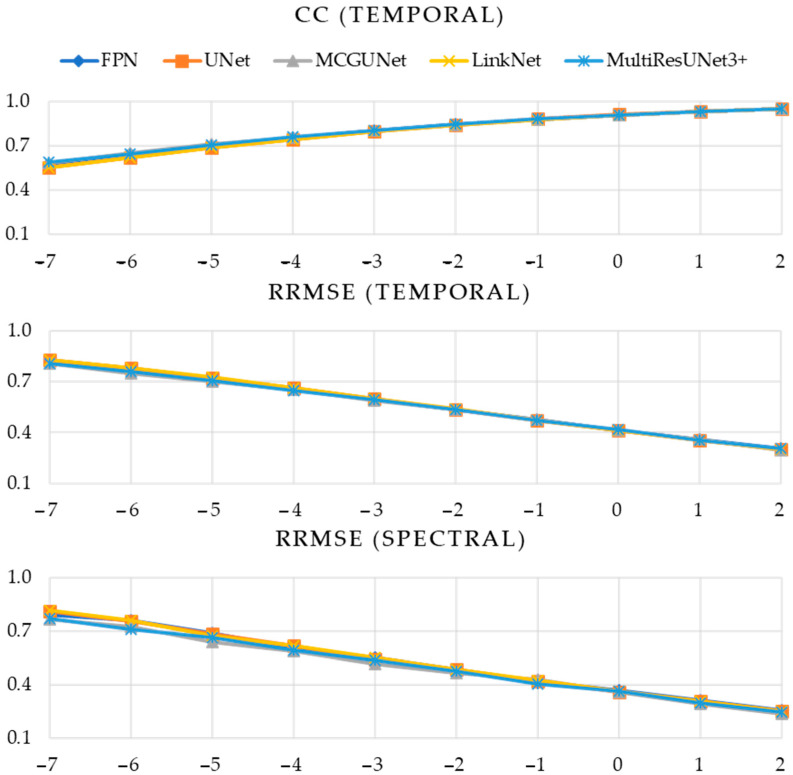
Estimated performance metrics (CC in time domain, RRMSE in time, and frequency domain vs. SNR) of five DL models after removing EMG artifacts from EMG-contaminated EEG.

**Figure 10:**
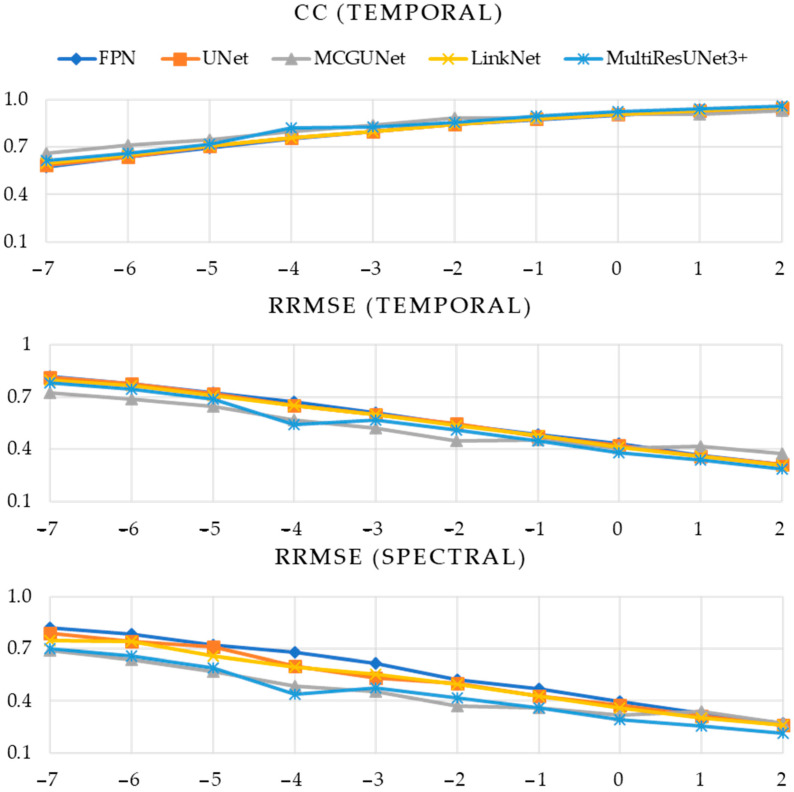
Estimated performance metrics (CC in time domain, RRMSE in time, and frequency domain vs. SNR) of five DL models after denoising simultaneous EOG and EMG-contaminated EEG.

In Table 1, Table 2 and Table 3, the numerical values of the three performance metrics (${CC}_{temporal}$, ${RRMSE}_{temporal}$, and ${RRMSE}_{spectral}$) are presented for the denoised EOG, EMG, and simultaneous EOG and EMG-contaminated EEG segments, respectively. On the other hand, Figure 8 illustrates the evaluation of five different 1D-CNN models in the context of EOG-contaminated EEG denoising. Specifically, the figure presents ${CC}_{temporal}$ values obtained across ten integer SNR levels, spanning from −7 to +2 dB. Additionally, Figure 8 also displays the ${RRMSE}_{temporal}$ and ${RRMSE}_{spectral}$ values plotted against the same ten SNR levels. Similarly, Figure 9 and Figure 10 illustrate the same performance metrics plotted against ten different SNR levels computed for the predicted denoised EMG-contaminated EEG segments and simultaneous EOG and EMG-contaminated EEG segments.

It is apparent from Table 1 and Figure 8 that our proposed MultiResUNet3+ model outperformed all remaining four 1D-segmentation models in terms of removing EOG artifacts from noisy EEG segments. This superiority is consistently observed across all ten different SNR levels. Specifically, the MultiResUNet3+ model achieved the highest CC values, indicating a higher correlation between the predicted and ground truth EEG segments. Furthermore, the RRMSE values obtained from the MultiResUNet3+ model were the lowest in both the temporal and spectral domains, again demonstrating its superior accuracy and precision in removing EOG artifacts. Overall, these findings highlight the effectiveness and robustness of the proposed MultiResUNet3+ model in reducing EOG artifacts from EOG-contaminated EEG denoising, even under challenging conditions characterized by low SNR levels.

As can be observed from Figure 9, the five 1D-CNN models displayed similar performance in removing EMG artifacts from the EMG-contaminated EEG segments, as indicated by the almost overlapping curves of all three-performance metrics. Given the difficulty of determining the best-performing model solely based on Figure 9, Table 2 is included to provide numerical values of ${CC}_{temporal}$, ${RRMSE}_{temporal}$, and ${RRMSE}_{spectral}$. The tabulated data highlight the inconsistent performance of the 1D-segmentation networks in predicting EMG-artifacts-free EEG. Notably, the proposed MultiResUNet3+ and MCGUNet demonstrated relatively superior performance compared to the other three models.

During the denoising of simultaneous EOG and EMG artifacts from corrupted EEG segments, the greatest temporal correlation coefficient (a little above 0.95) is obtained from MultiResUNet3+ for the segments having the lowest noise level (SNR level of +2 dB). The smallest RRMSE in the time and frequency domain is also achieved by our proposed MultiResUNet3+ model compared to the other four 1D-CNN models while estimating the performance on test segments (refer to Table 3 and Figure 10).

Overall, for EOG/EMG/simultaneous EOG and EMG artifacts removal, the efficacy of the deep learning (DL) models is observed to be enhanced in parallel with the increment of the SNR, as anticipated. The increase of SNR level results in a proportional reduction in the noise quotient (comprising EOG, EMG, and simultaneous EOG and EMG), thereby possibly reducing the complexity of the nonlinear mapping function acquired by the DL-based segmentation models for forecasting denoised EEG segments. Ultimately, this leads to an improvement in the model’s performance in predicting noise-free EEG.

### 4.2. Experiment B Outcomes

All five 1D-CNN models’ objective is to estimate artifacts-free EEG segments. To determine the most effective 1D-segmentation model in reducing physiological artifacts from corrupted EEG, five different performance metrics have been computed, namely (i) Temporal percentage reduction in artifacts (η), (ii) spectral percentage reduction in artifacts (γ), (iii) RRMSE in the time domain (${RRMSE}_{temporal}$), (iv) RRMSE in the frequency domain (${RRMSE}_{spectral}$), and (v) the average power ratio for five distinct EEG bands (Delta, Theta, Alpha, Beta, Gamma) for ground truth, noisy, and predicted artifacts-free EEG segments. Moreover, a sample corrupted, ground truth, and predicted clean EEG segment plots are provided for visual or qualitative assessment.

**Figure 11:**
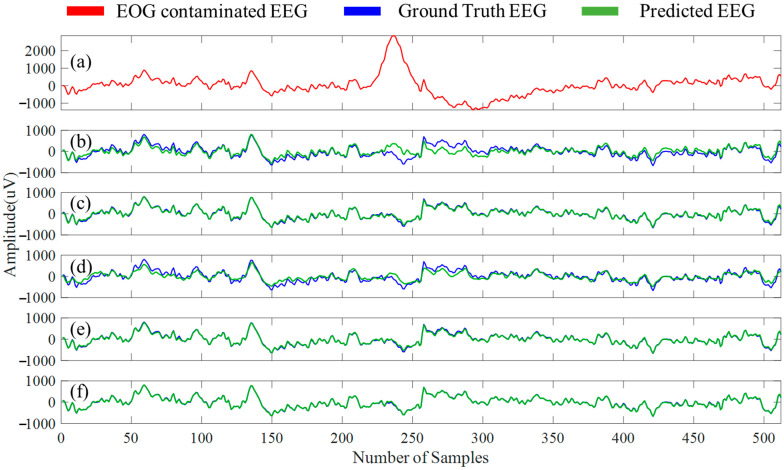
(**a**) A sample EOG-corrupted EEG segment, EOG artifacts-free EEG segment predicted by (**b**) FPN, (**c**) UNet, (**d**) MCGUNet, (**e**) LinkNet and (**f**) MultiResUNet3+ models plotted against the ground truth EEG.

Figure 11a depicts an EEG segment that has been contaminated with EOG artifacts, while Figure 11b–f illustrates EOG artifact-free EEG segments (estimated clean EEG) for all five models. Table 4 provides the average numerical values of the four performance metrics ($\eta ,\;\gamma ,\;{RRMSE}_{temporal},\;and\;{RRMSE}_{spectral}$) by the five distinct models. In contrast, Table 5 illustrates the computed average power ratios for five separate EEG bands for ground truth, noisy, and predicted EEG segments.

From Table 4, our proposed MultiResUNet3+ performed best in reducing EOG artifacts from EOG-contaminated EEG segments in the temporal and spectral domains (94.82% and 92.84%, respectively). Although the UNet model produced the lowest ${RRMSE}_{temporal}$ (~0.11) and ${RRMSE}_{spectral}$ (~0.12), respectively, MultiResUNet3+ was also very close. UNet produced the closest delta and alpha power ratio compared to the ground truth EEG. For theta, beta, and gamma band power ratio, our proposed MultiResUNet3+ performed best (refer to Table 5).

**Figure 12:**
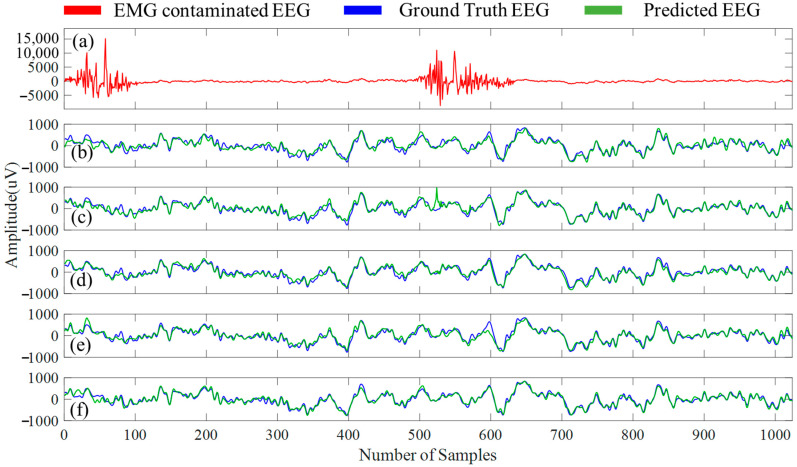
(**a**) An example EMG-corrupted EEG segment, EMG artifacts-free EEG segment predicted by (**b**) FPN, (**c**) UNet, (**d**) MCGUNet, (**e**) LinkNet, and (**f**) MultiResUNet3+ networks along with the ground truth EEG.

An example EMG-contaminated EEG segment is illustrated in Figure 12a, and to provide a qualitative overview of the five denoising 1D-CNN models, Figure 12b–f shows EMG artifacts-free EEG (predicted EEG) segments. Table 6 summarizes the four performance metrics ($\eta ,\;\gamma ,\;{RRMSE}_{temporal},\;and\;{RRMSE}_{spectral}$) obtained for the predicted EMG artifacts-free EEG segments by all the five 1D-CNN models separately, and Table 7 contains the average power ratio calculated for five different EEG bands before and after the removal of myogenic artifacts.

For EMG-corrupted EEG segments denoising, the proposed MultiResUNet3+ eliminated 83.21% of spectral EMG artifacts, whereas the UNet eliminated 89.59% of time domain EMG artifacts from the corrupted EEG segments. In terms of η, UNet performed best, and in terms of γ reduction, our proposed model performed best among all the models (refer to Table 6). Again, UNet and MultiResUNet3+ produced the lowest ${RRMSE}_{temporal}$ (~0.22) and ${RRMSE}_{spectral}$ (~0.19) values, respectively, when evaluated with the other four 1D CNN networks. Moreover, when comparing the power ratios computed using the MultiResUNet3+ model’s predicted segments with the ground truth EEG’s power ratios among the delta, beta, and gamma bands, the MultiResUNet3+ model comes out on top. For the theta and alpha band power ratio, LinkNet performed best (refer to Table 7).

**Figure 13:**
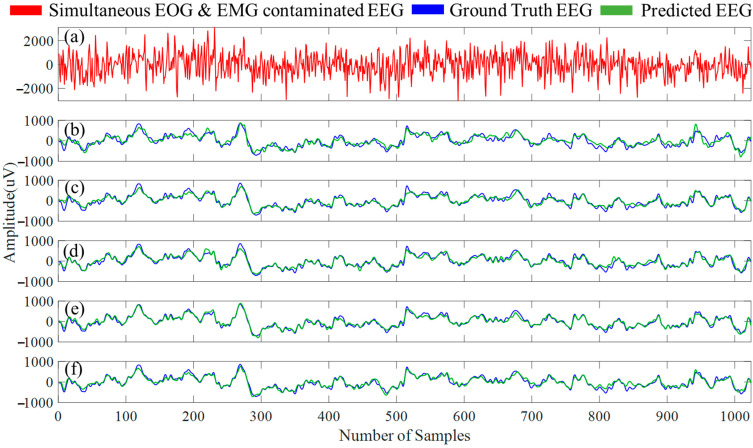
(**a**) An example simultaneous EOG and EMG-contaminated EEG segment. Simultaneous EOG and EMG-artifacts-free EEG segments created by (**b**) FPN, (**c**) UNet, (**d**) MCGUNet, (**e**) LinkNet, and (**f**) multiResUNet3+ networks along with the ground truth EEG.

In Figure 13a, an example segment of simultaneous EOG and EMG contaminated EEG segment is presented, and in Figure 13b–f, the denoised EEG segment (simultaneous EOG and EMG artifacts-free segment) along with the ground truth EEG is shown separately for the five 1D-CNN models. The four performance metrics for the five 1D-CNN models’ predictions on the denoised simultaneous EOG and EMG-contaminated EEG segments can be found in Table 8, whereas Table 9 provides the average power ratio between five different EEG bands before and after removing simultaneous EOG and EMG artifacts.

As observed from Table 8, the proposed MultiResUNet3+, UNet, and LinkNet models produced very close denoising performance for simultaneous EOG and EMG corrupted EEG segments. Artifacts are reduced by 89.77% in the time domain and 83.39% in the frequency domain when LinkNet and UNet models are used, respectively. When compared among the five 1D-CNN networks, the LinkNet model yielded the lowest ${RRMSE}_{temporal}$ (~0.22) and ${RRMSE}_{spectral}$ (~0.18) values. Finally, when compared to the ground truth EEG, the MultiResUNet3+ model excels in producing the nearest average power ratios for the delta band. However, the LinkNet model performed best in the theta, beta, and gamma band power ratio, whereas the FPN model showed superior performance in the alpha band (Table 9).

## 5. Discussion

The limitations of traditional single-stage and two-stage signal processing-based methods for denoising EEG signals, including low correlation coefficient, potential loss of crucial neural information, poor performance in dynamic situations, etc., are mentioned in the introduction section of this study. Although a few deep learning-based approaches have been presented for EEG denoising to remedy these drawbacks, with notable performance gains over signal processing-based methods, these DL-based approaches still have some shortcomings, among which lackings in model robustness, reliability, and generalizability are the key.

To address these limitations, five distinct DL models are utilized in this extensive study, including the proposed novel MultiResUNet3+ and four additional 1D-CNN models (FPN, UNet, MCGUNet, and LinkNet) to denoise EOG/EMG/simultaneous EOG and EMG artifacts from corrupted EEG. A publicly available dataset, namely EEGdenoiseNet, is deemed suitable for this work, as it featured pre-processed, well-structured EEG segments, including 4514 clean EEG segments, 3400 pure electrooculogram segments, and 5598 clean electromyogram segments. We semi-synthetically generated a large set of noisy EEG segments using linear mixing techniques where these clean EOG and EMG segments were used as noisy signals and clean EEG segments as ground truth to train the DL-based models. This process provided a broad range of signal types for the DL models to learn from. Furthermore, due to linear mixing, ground truth EEG segments were also available to provide a reliable reference for evaluating the performance of the models. To prevent any data leaking issues between the train and test set, the clean EEG, EOG, and EMG segments were used only once during the semi-synthetic data generation process.

Further, the quantitative measurement of five performance metrics (Experiment B) for all the five deep learning networks (FPN, UNet, MCGUNet, LinkNet, and the proposed novel MultiResUNet3+) showed that it is possible to denoise EEG signal artifacts using DL-based techniques and has a great potential to eliminate multiple artifacts simultaneously from EEG signals by using robust DL models. It should be added here that, for DL networks, high-frequency artifacts, such as EMG artifacts and simultaneous EOG and EMG artifacts, are more challenging to handle than low-frequency artifacts, such as EOG artifacts. The F-principle of neural networks can be used to explain this occurrence [66], which states that DL networks frequently learn low-frequency information at the beginning of training and high-frequency information as training iterations rise. A similar phenomenon is also observed in this study. From our extensive experiments, we have observed that removing EOG artifacts (low-frequency noise) from corrupted EEG is less challenging than denoising EMG-contaminated (high-frequency noise) EEG and simultaneous EOG and EMG-contaminated (high-frequency noise) EEG. For instance, a higher temporal percentage reduction in EOG artifacts (94.82%) is found than the temporal percentage reduction in EMG artifacts (89.59%). Similarly, a higher spectral percentage reduction in EOG artifacts (92.84%) is computed than in EMG artifacts (83.21%). Although simultaneous EOG and EMG contamination in EEG is very unlikely in a real-life scenario and challenging to remove reliably, our proposed MultiResUNet3+ provided a staggering performance—clear evidence of the efficacy and superiority of our proposed model in denoising complex artifacts as well.

Although the DL-based denoising approaches need a vast amount of ground truth EEG data during the training phase, once the model is trained, it can be utilized reliably to eradicate artifacts from unseen EEG signals corrupted with physiological artifacts. Another benefit of DL models is the ability to handle complicated artifact mixes, such as nonlinear and stationary ones. In contrast to the traditional signal processing approaches, which typically linearly attenuate artifacts, DL models can directly learn the fundamental pattern of neural activities from training data in the hidden space and then synthesize/predict the clean EEG from corrupted EEG. Therefore, DL-based techniques perform better than conventional methods in removing physiological noises from contaminated EEG.

It is indeed worth mentioning some constraints of this study. Even though the semi-synthetic EEG dataset generated in this study includes a very high number of clean EEG, EOG, and EMG segments, the lesser variability in EEG type and lack of diversity in artifacts type is a matter of concern since EEG data can be recorded when the subject is at rest or while doing various tasks. Moreover, artifacts in EEG recordings are not just limited to ocular and myogenic. Motion artifacts, one of the inevitable sources of non-physiological noise, become dominant while EEG data are captured via wearable sensors. Therefore, a more diverse and dynamic dataset curation is necessary, which will help the DL-based models to be more efficient in removing physiological and non-physiological artifacts from corrupted EEG signals.

## 6. Conclusions

The elimination of artifacts is a crucial aspect of EEG data analysis. In this paper, we presented a novel 1D-CNN model, i.e., MultiResUNet3+ for EEG denoising, and demonstrated its superiority over four other 1D-CNN models, namely FPN, UNet, MCGUNet, and LinkNet. Apart from proposing a novel 1D-CNN model for denoising EEG reliably, we also introduced a set of benchmark metrics to facilitate the quantitative evaluation of DL-based EEG denoising models’ adeptness. The proposed MultiResUNet3+ model reduced the EOG artifacts from EOG-corrupted EEG by 94.82% and 92.84% in the time and frequency domains, respectively. Moreover, the nearest average power ratio for the theta, beta, and gamma bands (0.5295, 0.076, 0.0197) compared with the ground truth EEG (0.5184, 0.0749, 0.0195) while removing the EOG artifact was produced by MultiResUNet3+, meaning our proposed model is capable of denoising EEG in those bands most accurately. For EMG artifacts removal from contaminated EEG, our proposed novel MultiResUNet3+ performed best by reducing 83.21% artifacts in the frequency domain and a neck-to-neck 89.33% artifacts reduction in the time domain (the best is 89.59%, produced by UNet). While denoising EMG-contaminated EEG segments, MultiResUNet3+ had the lowest spectral RRMSE (0.1893) as well. Once EMG artifacts were removed from the corrupted EEG segments, the proposed MultResUNet3+ provided the nearest average power ratio in delta, beta, and gamma bands (0.4453, 0.0658, and 0.0165, respectively) compared with the ground truth EEG (0.4421, 0.0756, and 0.0197, respectively). In denoising, simultaneous EOG and EMG from corrupted EEG, three 1D-CNN models, namely UNet, LinkNet, and novel MultiResUNet3+, performed neck-to-neck, which is evident from the computed performance evaluation metrics. Overall, the results obtained from the proposed MultiResUNet3+ clearly showed its robustness and reliability in denoising physiological artifacts (EOG/EMG/simultaneous EOG and EMG) from corrupted EEG signals. Our findings showed that DL techniques could potentially eliminate EOG/EMG artifacts from EEG data even at high noise levels. Following a similar framework, the proposed MultiResUNet3+ segmentation network may be used for multi-channel EEG noise reduction by applying the proposed model to multi-channel EEG signals separately, which should facilitate EEG-based BCI applications.

## Figures and Tables

**Table 1:** EOG-contaminated EEG segment denoising performance. The bold numeric values represent the best performance.

Performance Metric	SNR (dB)	Model				
		FPN	UNet	MCGUNet	LinkNet	MultiResUNet3+ (Proposed)
CC (Temporal)	−7	0.8074	0.8200	0.7913	0.8269	**0.8636**
	−6	0.8320	0.8507	0.8308	0.8566	**0.8865**
	−5	0.8522	0.8646	0.8459	0.8579	**0.9032**
	−4	0.8695	0.8912	0.8506	0.8793	**0.9152**
	−3	0.8819	0.8986	0.8597	0.8946	**0.9329**
	−2	0.9004	0.9299	0.8617	0.9204	**0.9347**
	−1	0.9136	0.9389	0.8710	0.9261	**0.9437**
	0	0.9286	0.9420	0.8783	0.9388	**0.9534**
	1	0.9322	0.9442	0.8844	0.9494	**0.9633**
	2	0.9493	0.9554	0.8948	0.9537	**0.9644**
RRMSE (Temporal)	−7	0.5903	0.5693	0.5733	0.5554	**0.4694**
	−6	0.5567	0.5146	0.5378	0.5098	**0.4279**
	−5	0.5184	0.4970	0.4982	0.5146	**0.3954**
	−4	0.4939	0.4377	0.4942	0.4793	**0.3728**
	−3	0.4739	0.4341	0.4768	0.4416	**0.3193**
	−2	0.4357	0.3386	0.4785	0.3735	**0.3232**
	−1	0.3949	0.3208	0.4698	0.3662	**0.3021**
	0	0.3552	0.3134	0.4630	0.3256	**0.2674**
	1	0.3501	0.3073	0.4584	0.2840	**0.2413**
	2	0.2907	0.2666	0.4197	0.2744	**0.2338**
RRMSE (Spectral)	−7	0.6219	0.5854	0.5066	0.5677	**0.4419**
	−6	0.5827	0.5278	0.4807	0.5328	**0.4234**
	−5	0.5601	0.5280	0.4372	0.5514	**0.3948**
	−4	0.5471	0.4492	0.4048	0.5331	**0.3827**
	−3	0.5164	0.4754	0.4022	0.4687	**0.3219**
	−2	0.4902	0.3605	0.3992	0.3996	**0.3382**
	−1	0.4354	0.3498	0.3849	0.4131	**0.3224**
	0	0.3834	0.3471	0.3615	0.3621	**0.2878**
	1	0.3897	0.3384	0.3584	0.3134	**0.2707**
	2	0.3168	0.2992	0.3207	0.3080	**0.2668**

**Table 2:** EMG-contaminated EEG segment denoising performance where the bold numeric values represent the best performance.

Performance Metric	SNR (dB)	Model				
		FPN	UNet	MCGUNet	LinkNet	MultiResUNet3+ (Proposed)
CC (Temporal)	−7	0.5779	0.5548	0.5862	0.5552	**0.5897**
	−6	0.6425	0.6198	**0.6533**	0.6224	0.6463
	−5	0.7013	0.6879	**0.7095**	0.6864	0.7056
	−4	0.7545	0.7447	0.7561	0.7448	**0.7589**
	−3	0.8015	0.7992	0.8035	0.7973	**0.8039**
	−2	0.8438	0.8418	0.8410	0.8417	**0.8447**
	−1	0.8785	0.8803	0.8793	0.8793	**0.8826**
	0	0.91	**0.9113**	0.9088	0.9106	0.9092
	1	0.9336	**0.9345**	0.9336	0.9343	0.9343
	2	0.9517	0.9529	0.9519	0.9526	**0.9536**
RRMSE (Temporal)	−7	0.8136	0.8266	0.8048	0.8263	**0.8049**
	−6	0.7672	0.7813	**0.7486**	0.7792	0.7597
	−5	0.7119	0.7215	**0.6994**	0.7249	0.7087
	−4	0.6544	0.6654	0.6536	0.6643	**0.6483**
	−3	0.5963	0.6003	**0.5884**	0.6008	0.5924
	−2	0.5345	0.5378	0.5355	0.5387	**0.5333**
	−1	0.4775	0.4738	0.4746	0.4741	**0.4695**
	0	0.4130	**0.4102**	0.4151	0.4115	0.4158
	1	0.3570	**0.3548**	0.3571	0.3566	0.3565
	2	0.3055	0.3029	**0.2968**	0.3034	0.3058
RRMSE (Spectral)	−7	0.7920	0.8127	**0.7670**	0.8157	0.7686
	−6	0.7622	0.7540	0.7255	0.7581	**0.7087**
	−5	0.6905	0.6857	**0.6389**	0.6716	0.6643
	−4	0.5997	0.6180	**0.5870**	0.6162	0.5926
	−3	0.5512	0.5404	**0.5167**	0.5508	0.5344
	−2	0.4843	0.4836	**0.4664**	0.4825	0.4770
	−1	0.4206	0.4208	0.4283	0.4246	**0.4042**
	0	0.3685	0.3595	**0.3552**	0.3617	0.3615
	1	0.3115	0.3054	**0.2908**	0.3064	0.2971
	2	0.2544	0.2527	**0.2361**	0.2520	0.2479

**Table 3:** Simultaneous EOG and EMG-contaminated EEG segments denoising performance. The bold numeric values represent the best performance.

Performance Metric	SNR (dB)	Model				
		FPN	UNet	MCGUNet	LinkNet	MultiResUNet3+ (Proposed)
CC (Temporal)	−7	0.5771	0.5856	**0.6630**	0.5987	0.6152
	−6	0.6370	0.6355	**0.7113**	0.6512	0.6582
	−5	0.6932	0.7039	**0.7449**	0.7052	0.7188
	−4	0.7507	0.7565	0.8009	0.7576	**0.8191**
	−3	0.7982	0.8003	**0.8388**	0.8010	0.8245
	−2	0.8423	0.8428	**0.8819**	0.8431	0.8580
	−1	0.8757	0.8803	0.8832	0.8780	**0.8934**
	0	0.9025	0.9084	0.9094	0.9098	**0.9228**
	1	0.9308	0.9325	0.9100	0.9332	**0.9411**
	2	0.9496	0.9504	0.9277	0.9506	**0.9579**
RRMSE (Temporal)	−7	0.8198	0.8122	**0.7235**	0.7989	0.7830
	−6	0.7758	0.7755	**0.6873**	0.7638	0.7446
	−5	0.7247	0.7189	**0.6479**	0.7088	0.6883
	−4	0.6706	0.6524	0.5688	0.6532	**0.5440**
	−3	0.6108	0.6017	**0.5222**	0.5994	0.5675
	−2	0.5438	0.5458	**0.4476**	0.5370	0.5108
	−1	0.4871	0.4736	0.4526	0.4821	**0.4480**
	0	0.4338	0.4207	0.4054	0.4143	**0.3821**
	1	0.3664	0.3603	0.4183	0.3610	**0.3375**
	2	0.3159	0.3118	0.3744	0.3107	**0.2867**
RRMSE (Spectral)	−7	0.8202	0.7903	**0.6894**	0.7447	0.7017
	−6	0.7842	0.7429	**0.6364**	0.7399	0.6587
	−5	0.7222	0.7088	**0.5690**	0.6578	0.5879
	−4	0.6812	0.5999	0.4865	0.5969	**0.4401**
	−3	0.6136	0.5341	**0.4545**	0.5559	0.4768
	−2	0.5216	0.5027	**0.3698**	0.4972	0.4188
	−1	0.4701	0.4287	0.3625	0.4262	**0.3577**
	0	0.3945	0.3763	0.3196	0.3576	**0.2934**
	1	0.3289	0.3134	0.3374	0.3048	**0.2542**
	2	0.2716	0.2635	0.2722	0.2622	**0.2142**

**Table 4:** Measured performance metrics after denoising EOG-contaminated EEG where the bold numeric values represent the best performance.

Model	η (%)	γ (%)	${RRMSE}_{temporal}$ ± STD	${RRMSE}_{spectral}$ ± STD
FPN	88.26	81.31	0.23141 ± 0.07250	0.25518 ± 0.08122
UNet	94.59	91.59	**0.11342** ± **0.03825**	**0.12123** ± **0.04411**
MCGUNet	73.28	70.39	0.43087 ± 0.08775	0.43669 ± 0.13015
LinkNet	94.40	91.30	0.12072 ± 0.04134	0.13229 ± 0.04826
MultiResUNet3+ (Proposed)	**94.82**	**92.84**	0.13190 ± 0.05364	0.13660 ± 0.05587

**Table 5:** Estimated power ratios of five distinct EEG frequency bands for the ground truth, noisy, and predicted EOG-artifacts-free EEG segments. The bold numeric values represent the best performance.

Model/Method	Delta	Theta	Alpha	Beta	Gamma
FPN	0.4147	0.5429	0.1278	0.0797	0.0208
UNet	**0.4338**	0.5297	**0.1230**	0.0761	0.0200
MCGUNet	0.3997	0.6057	0.1289	0.0543	0.0101
LinkNet	0.4320	0.5318	0.1234	0.0762	0.0199
MultiResUNet3+ (Proposed)	0.4337	**0.5295**	0.1233	**0.0760**	**0.0197**
EOG-contaminated EEG	0.8301	0.1639	0.0368	0.0244	0.0069
Ground Truth EEG	0.4459	0.5184	0.1206	0.0749	0.0195

**Table 6:** Measured performance metrics after denoising EMG-contaminated EEG. The bold numeric values represent the best performance.

Model	η (in %)	γ (in %)	${RRMSE}_{temporal}$ ± STD	${RRMSE}_{spectral}$ ± STD
FPN	85.06	75.96	0.31727 ± 0.11152	0.26395 ± 0.11695
UNet	**89.59**	80.61	**0.22394** ± **0.07785**	0.19231 ± 0.08107
MCGUNet	87.19	79.37	0.29214 ± 0.10520	0.23798 ± 0.10421
LinkNet	88.94	82.60	0.25091 ± 0.10112	0.19961 ± 0.10910
MultiResUNet3+ (Proposed)	89.33	**83.21**	0.23769 ± 0.08841	**0.18931** ± **0.08931**

**Table 7:** Power ratios (estimated) of five distinct EEG frequency bands for the ground truth, noisy, and predicted EMG-artifacts-free EEG. The bold numeric values represent the best performance.

Model/Method	Delta	Theta	Alpha	Beta	Gamma
FPN	0.4500	0.5534	0.1115	0.0566	0.0125
UNet	0.4452	0.5452	0.1150	0.0612	0.0147
MCGUNet	0.4393	0.5436	0.1193	0.0648	0.0155
LinkNet	0.4480	**0.5303**	**0.1195**	0.0656	0.0162
MultiResUNet3+ (Proposed)	**0.4453**	0.5344	0.1178	**0.0658**	**0.0165**
EMG contaminated EEG	0.1333	0.1046	0.0622	0.2079	0.5514
Ground Truth EEG	0.4421	0.5163	0.1230	0.0756	0.0197

**Table 8:** Measured performance metrics after denoising simultaneous EOG and EMG-contaminated EEG. The bold numeric values represent the best performance.

Model	η (in %)	γ (in %)	${RRMSE}_{temporal}$ ± STD	${RRMSE}_{spectral}$ ± STD
FPN	84.19	77.97	0.34448 ± 0.13849	0.27145 ± 0.15104
UNet	89.63	**83.63**	0.22991 ± 0.09619	0.18805 ± 0.10819
MCGUNet	87.03	80.31	0.29993 ± 0.10971	0.25156 ± 0.11219
LinkNet	**89.77**	83.39	**0.22439** ± **0.09180**	**0.18340** ± **0.10208**
MultiResUNet3+ (Proposed)	89.16	82.71	0.24891 ± 0.09445	0.20085 ± 0.09989

**Table 9:** Estimated power ratios of five distinct EEG frequency bands for the ground truth, noisy, and predicted simultaneous EOG and EMG-artifacts-free EEG segments. The bold numeric values represent the best performance.

Model/Method	Delta	Theta	Alpha	Beta	Gamma
FPN	0.4543	0.5332	**0.1185**	0.0647	0.0153
UNet	0.4537	0.5304	0.1178	0.0650	0.0158
MCGUNet	0.4543	0.5443	0.1141	0.0595	0.0143
LinkNet	0.4537	**0.5288**	0.1175	**0.0659**	**0.0163**
MultiResUNet3+ (Proposed)	**0.4532**	0.5416	0.1131	0.0608	0.0151
Simultaneous EOG-EMG contaminated EEG	0.1355	0.1046	0.0614	0.0196	0.5503
Ground Truth EEG	0.4458	0.5180	0.1208	0.0749	0.0196

## Data Availability

Zhang et al. [50] generously provided the data utilized in this study, and the semi-synthetically generated EEG dataset can be accessed by the corresponding authors of this work by making a reasonable request.
